# Asymmetric affective forecasting errors and their correlation with subjective well-being

**DOI:** 10.1371/journal.pone.0192941

**Published:** 2018-03-07

**Authors:** Marco Bertoni, Luca Corazzini

**Affiliations:** 1 Department of Economics and Management, University of Padova, Padova, Italy; 2 Department of Economic Sciences, University of Venice “Cà Foscari”, Venice, Italy; University of Zurich, SWITZERLAND

## Abstract

**Aims:**

Social scientists have postulated that the discrepancy between achievements and expectations affects individuals' subjective well-being. Still, little has been done to qualify and quantify such a psychological effect. Our empirical analysis assesses the consequences of positive and negative affective forecasting errors—the difference between realized and expected subjective well-being—on the subsequent level of subjective well-being.

**Data:**

We use longitudinal data on a representative sample of 13,431 individuals from the German Socio-Economic Panel. In our sample, 52% of individuals are females, average age is 43 years, average years of education is 11.4 and 27% of our sample lives in East Germany. Subjective well-being (measured by self-reported life satisfaction) is assessed on a 0–10 discrete scale and its sample average is equal to 6.75 points.

**Methods:**

We develop a simple theoretical framework to assess the consequences of positive and negative affective forecasting errors—the difference between realized and expected subjective well-being—on the subsequent level of subjective well-being, properly accounting for the endogenous adjustment of expectations to positive and negative affective forecasting errors, and use it to derive testable predictions. Given the theoretical framework, we estimate two panel-data equations, the first depicting the association between positive and negative affective forecasting errors and the successive level of subjective well-being and the second describing the correlation between subjective well-being expectations for the future and hedonic failures and successes. Our models control for individual fixed effects and a large battery of time-varying demographic characteristics, health and socio-economic status.

**Results and conclusions:**

While surpassing expectations is uncorrelated with subjective well-being, failing to match expectations is negatively associated with subsequent realizations of subjective well-being. Expectations are positively (negatively) correlated to positive (negative) forecasting errors. We speculate that in the first case the positive adjustment in expectations is strong enough to cancel out the potential positive effects on subjective well-being of beaten expectations, while in the second case it is not, and individuals persistently bear the negative emotional consequences of not achieving expectations.

“The less you expect, the more you'll be pleased”

Lyrics from the song “*Less*”, Ben Harper & The Innocent Criminals, 1999.

## Introduction

Expectations do matter in life. They represent a reference according to which individuals evaluate their economic, social and psychological conditions, and make important prospective decisions [1]. For instance, expectations and aspirations play a crucial role in determining investment in education [2,3], consumption choices [4], bequests decisions [5], and long-term career choices [6].

By modifying how experiences and subjective conditions are framed and contextualized [7–9], expectations may also affect subjective well-being judgments. Becoming aware of the discrepancy between what an individual achieved and her initial expectations may change the way in which she interprets life events, evaluates her subjective conditions and revises her life plans [10–13]. For instance, Sen [8,9] argues that the experience of negative conditions may push individuals to adjust their desires to contextual constraints, thereby “deforming” their expectations in response to realized life events.

The aim of our contribution is to investigate the consequences that the discrepancy between expectations and realizations of subjective well-being exerts on its subsequent level.

We depict a simple and stylized model of subjective well-being formation relying on a set of well-known economic and psychological assumptions, that will be amply discussed in the next pages. Net of the effects of both economic (such as labor market status and income) and non-economic (including socio-demographic and health conditions) factors [14–18], subjective well-being also depends on how individuals perceive to have performed relative to some initial expectation. Individuals suffer a loss of well-being from realizing to have failed to achieve what initially expected, while they enjoy an extra source of well-being in case they realize to have outperformed their target [19–21]. The extra gains and losses of well-being produced by the discrepancy between realizations and expectations depends on two additional elements. First, individuals might conceive achievements and failures differently, being more sensitive and assigning higher importance to either one of the two outcomes [22,23]. Second, expectations are not static, but instead adjust along with achievements and failures: they are revised up in case individuals have outperformed, while they are revised down in case individuals have failed to reach their initial expectations [24,25].

Our empirical analysis addresses three relevant research questions that are related to the assumptions and implications of our model and which, in our opinion, are still open in social sciences.

### Q.1. Is there any association between affective forecasting errors and subjective well-being?

Wilson and Gilbert [26] define *affective forecasting* as people’s predictions about future feelings, and call affective forecasting *error* the observed discrepancy between expected and realized feelings.

Theoretical contributions postulate that affective forecasting errors impact on individuals’ subjective well-being [19–21]. For instance, Higgins’ self-discrepancy theory [27] postulates that emotional discomfort arises if the actual self does not match the ideal self. Still, little has been done to qualify and empirically quantify such a psychological effect. As pointed out by Wilson and Gilbert [26], “most of the early work (…) measured people’s forecast but not their actual emotional response” (p.346).

In order to fill this gap, we use microdata from the German Socio-Economic Panel (SOEP, [28]). This unique dataset is representative of the German population and collects longitudinal information on respondents’ characteristics, their expectations about future life satisfaction and the subsequent life satisfaction realizations. These two variables can thus be matched to compute affective forecasting errors about subjective well-being as the difference between realized and expected life satisfaction, allowing us to understand how positive or negative errors are correlated with future life satisfaction.

In a seminal paper based on the SOEP dataset, Schwandt [29] finds that people systematically mis-predict their subjective well-being over the life cycle: they expect–incorrectly–increases in young adulthood and decreases during old age. These findings provide supporting evidence for theories explaining the age U-shape in well-being with unmet expectations.

In line with the recent empirical literature in economics, psychology, and sociology, we measure subjective well-being with self-reported overall life satisfaction [14,25,30]. Therefore, we will henceforth consider the terms “subjective well-being” and “life satisfaction” as synonyms, and use them interchangeably.

Given these considerations, the first testable prediction we investigate is whether, even after controlling for individual fixed effects, a number of time-varying characteristics of respondents measured at the time of the expectation formulation, and their evolution up to the realization of life satisfaction, affective forecasting errors are correlated with individual subjective well-being. Thus, rather than contributing to the extensive literature on the determinants of faulty affective forecasting, we are mainly interested in estimating its net association with subsequent well-being.

It is worth noticing that we discuss our empirical results in terms of correlations–not of causal effects–because, even if we control for individual fixed effects, a number of time-varying characteristics of respondents measured at the time of the expectation formulation, and their evolution up to the realization of life satisfaction, there is still the possibility that negative unobservable shocks affecting well-being can be more persistent than positive ones, which would make individuals have both unmet expectations *and* lower life satisfaction in the next period, but not the other way around. This is a remaining empirical difficulty that we can hardly overcome. Still, in the robustness section we discuss a placebo exercise that shall help to dispel this concern.

Previous empirical studies mainly focus on the role played by income expectations in determining subjective well-being [12,22,31–35], and this approach has undoubtedly produced meaningful conclusions. For instance, it provides a simple and appealing explanation for the (almost) flat relationship between subjective well-being and the per-capita level of GDP that is empirically observed in developed nations [36,37].

Nevertheless, “money is not enough to make people happy” and, in addition to income, there are other important non-economic dimensions that play a crucial role in determining subjective well-being [14–18]. In this perspective, overall subjective well-being can be described as a weighted average of satisfaction with several economic and non-economic aspects of life [38]. Thus, as much as they do with income, it is reasonable to believe that individuals evaluate achievements relative to previous expectations about overall well-being, that are formulated by considering economic as well as non-economic life domains. To understand whether expectations about *income* or *non-income* related aspects of life are the relevant ones for well-being, in an extension to our analysis, we compare results of our main specification–that uses affective forecasting errors defined in terms of subjective well-being–and of a second one, that considers the well-being consequences of discrepancies between pay rise expectations and realized changes in labor income.

### Q.2. Does the association with subjective well-being change between positive and negative affective forecasting errors?

The magnitude of the psychological response to affective forecasting errors might depend on the sign of the discrepancy between actual conditions and expectations. The differential effects of achieving or missing expectation is central in the disappointment theory presented by Loomes and Sugden [23]. According to this theory, when facing an uncertain outcome, an individual formulates prior expectations on its realization. Then, after the uncertainty is resolved, the individual experiences an extra gain of utility (due to elation) if the outcome is better than what expected, while she bears an extra loss of utility (due to disappointment) if she finds her situation to be below the prior expectations. There is no reason to expect the two additional components of utility to be symmetric in size. Indeed, there is robust evidence suggesting that individuals are loss averse [22]: for a shock of given size, the loss in well-being registered when the shock is negative and, relative to an initial reference, associated with deteriorated conditions is greater than the gain in well-being when the shock is positive and associated with improved conditions.

Boyce at al. [39] study the association between income changes and subjective well-being. They show that income losses exert a larger effect on well-being than equivalent income gains, and conclude that loss aversion does not only represent an affective forecasting error [40] but also applies to experienced losses.

In our context, these considerations would suggest that “unmet” expectations (i.e. the level of life satisfaction achieved by the individual falls below her own expectations–a *negative* affective forecasting error) should be more strongly correlated with well-being levels than “beaten” expectations (i.e. the level of life satisfaction achieved by the individual overcomes her own expectations–a *positive* affective forecasting error).

### Q.3. Is there any empirical association between affective forecasting errors and subjective well-being expectations for the future?

Expectations (on future levels of subjective well-being) are not fixed, rather, they represent an endogenous reference that adjusts over time to life events, to smooth out the psychological responses to achievements or failures. Economists talk of reference-dependent preferences: the utility of a choice depends on the comparison between the corresponding outcome and the reference which, in turn, coincides with her (endogenous) equilibrium rational expectation about its level [41]. More related to the aim of this study, in discussing the “aspirations treadmill”, Kahneman and Kruger [25] postulate that “if people gradually adjust their aspirations to the utility that they normally experience, an improvement of life circumstances would eventually lead them to report no higher life satisfaction than they did before, even if they were experiencing higher utility than previously” (p.16). Similarly, Easterlin [24] posit that an increase in income leads to small and transitory improvements of life satisfaction, because income aspirations move in parallel with income levels. Sometimes, social scientists refer to this psychological adjustment process with the expression “preferences drift” [42].

Supporters of hedonic adaptation have provided robust evidence showing that individuals adapt faster to improved conditions than to unfavorable circumstances [43,44,45,7]. In our analysis, we explicitly consider whether there is any correlation between surpassed and unmet past expectations about life satisfaction and subjective well-being expectations for the future.

## Theoretical framework

Let *S*_*t*_ be individual’s realized life satisfaction for period t. Deaton [10] argues that the evaluation of subjective well-being is a relative one, as people compare their situation with a subjective benchmark, a “shifting standard” that depends on one’s expectations and past experiences. We follow this suggestion and postulate that realized life satisfaction depends on its latent dimension—$S_{t}^{*}$—and on the affective forecasting error $S_{t}^{*}-E_{t-1}(S_{t})$ according to the following equation:

$$S_{t}=S_{t}^{*}+\delta^{+}1[S_{t}^{*}-E_{t-1}(S_{t})>0]+\delta^{-}1[S_{t}^{*}-E_{t-1}(S_{t})<0].$$ (1)

In Eq 1, *δ*^+^ and *δ*^−^ are a positive and a negative coefficient that can be of different absolute magnitude: our specification allows positive and negative affective forecasting errors to affect realized satisfaction differently, in line with prospect theory and the idea of loss aversion. For simplicity and consistency with our empirical analysis, we consider simple indicator functions for positive and negative affective forecasting errors, but it would make no qualitative difference if they entered linearly in the model.

The remaining ingredients of our theoretical model are the specifications for latent life satisfaction—$S_{t}^{*}$—and for life satisfaction expectations– *E*_*t*−1_(*S*_*t*_).

Simplifying the formulation proposed by Schwandt [29], we let latent satisfaction $S_{t}^{*}$ be described as follows:

$$S_{t}^{*}=g(x_{t}){+\phi }^{+}1[S_{t-1}-E_{t-2}(S_{t-1})>0]+\phi^{-}1[S_{t-1}-E_{t-2}(S_{t-1})<0].$$ (2)

As in Schwandt [29], satisfaction depends on a set of (observable and unobservable) characteristics of individuals, summarized by *g*(*x*_*t*_). Additionally, we posit that individuals suffer or gain satisfaction if–in the previous period–they enjoyed a lower or higher level of satisfaction than they expected. Even in this case, we allow for asymmetric effects of positive and negative forecasting errors on latent satisfaction (*ϕ*^+^ and *ϕ*^−^). For simplicity, and unlike Schwandt [29], we exclude previous affective forecasting errors from the determination of latent satisfaction at time *t*. It is worth noticing that we treat differently affective forecasting errors at *t-1* and at *t* in the determination of satisfaction. In fact, our framework is based on the assumption that the former affects latent satisfaction directly, while the latter matters only once latent satisfaction judgments are formulated and individuals finalize their evaluation of satisfaction by comparing their current latent status with their expectations about it, as described in Eq 1.

Finally, as done by Schwandt [29], we also assume that expectations formulated at time *t-1* about life satisfaction at time *t* are a function of satisfaction at the time of the forecast—*S*_*t*−1_–but we assume that that they also depend on the affective forecasting error realized in the previous period. That is to say, individuals learn from their past affective forecasting errors to when updating their expectations about the future. Again, we allow for positive and negative affective forecasting errors to matter asymmetrically in affecting expectation updating (*β*^+^and *β*^−^). Hence, we posit the following specification for *E*_*t*−1_(*S*_*t*_):

$$E_{t-1}(S_{t})=\omega S_{t-1}+\beta^{+}1[S_{t-1}-E_{t-2}(S_{t-1})>0]+\beta^{-}1[S_{t-1}-E_{t-2}(S_{t-1})<0]$$ (3)

The solution of the model is obtained by substituting Eq (2) and Eq (3) into Eq (1). If we do so, we obtain the following specification for *S*_*t*_:

$$S_{t}=g(x_{t}){+\phi }^{+}1[S_{t-1}-E_{t-2}(S_{t-1})>0]+\phi^{-}1[S_{t-1}-E_{t-2}(S_{t-1})<0]$$

$$+\delta^{+}\left[\begin{array}{c} g(x_{t}){+\phi }^{+}1[S_{t-1}-E_{t-2}(S_{t-1})>0]+\phi^{-}1[S_{t-1}-E_{t-2}(S_{t-1})<0]\\ -\omega S_{t-1}-\beta^{+}1[S_{t-1}-E_{t-2}(S_{t-1})>0]-\beta^{-}1[S_{t-1}-E_{t-2}(S_{t-1})<0] \end{array}\right]>0$$ (4)

$$+\delta^{-}\left[\begin{array}{c} g(x_{t}){+\phi }^{+}1[S_{t-1}-E_{t-2}(S_{t-1})>0]+\phi^{-}1[S_{t-1}-E_{t-2}(S_{t-1})<0]\\ -\omega S_{t-1}-\beta^{+}1[S_{t-1}-E_{t-2}(S_{t-1})>0]-\beta^{-}1[S_{t-1}-E_{t-2}(S_{t-1})<0] \end{array}\right]>0.$$

In Eq (4), positive and negative affective forecasting errors at time *t-1* influence realized life satisfaction at time *t* by three channels:

1. First, as shown by the first line of Eq 4 they have a direct impact by the effect of the mismatches onto current latent life satisfaction (Eq 2).
2. Second, the effect of the mismatches onto current latent life satisfaction (Eq 2) also comes into play in the two terms that describe the comparison between latent and expected satisfaction at time t, pre-multiplied by *δ*^+^ or *δ*^−^, pictured in the second and third line of Eq 4.
3. Third, affective forecasting errors matter in the comparison between latent and expected satisfaction at time *t* not only because they influence $S_{t}^{*}$, but also because they are used to update expectations *E*_*t*−1_(*S*_*t*_).

In our empirical analysis, we will estimate two sets of parameters. First, we will estimate the overall effects of positive and negative affective forecasting errors at time *t-1* onto realized life satisfaction at time *t*, that is, a stripped-down version of Eq 4. Second, we will estimate how expectations about future life satisfaction formulated at time *t-1*, when the affective forecasting errors are realized, are affected by these forecasting errors, as portrayed by Eq 3. Needless to say, we cannot aim at estimating Eq 1 or Eq 2, as they involve the latent–and unobservable–term, $S_{t}^{*}$. Hence, we will not be able to distinguish empirically between the first two channels depicted by the model.

## Methods

### The data

#### The German Socio-Economic Panel (SOEP)

The SOEP is a representative annual panel survey of the German population, interviewing every year around 7,000 households (13,000 individuals). It started in 1984 in West Germany and in 1990, after German re-unification, in East Germany. The SOEP collects a wealth of information about subjective well-being: individuals are asked every year about their current satisfaction with many life domains (health, income, leisure, …) and about satisfaction with life in general. Our analysis is based on the Socio-Economic Panel (SOEP), data for years 1984–2012, version 29, SOEP, 2013 (doi: 10.5684/soep.v29). The SOEP is approved as being in accordance with the standards of the Federal Republic of Germany for lawful data protection, and all participants gave free and informed consent to participate in the survey. The survey ethics are monitored by an independent advisory board at the DIW—Berlin. The authors (who are not affiliated to DIW) signed a contract with the data holders to permit the use and publishing of data for scientific purposes. Interested researchers can access the data conditional on application. Details about the application process can be found at https://www.diw.de/en/diw_02.c.222829.en/access.html

#### Life satisfaction and affective forecasting errors

The unique feature of this dataset is that, from 1991 until 2004, individuals were also consistently and repeatedly asked about their expected life satisfaction in five years’ time. The exact wording of the questions in English is as follows: “*How satisfied are you at present with your life as a whole*?*”* and *“How satisfied do you think you will be five years from now*?*”*. Individuals were asked to report their answer according to a 0–10 scale, where 0 means 'completely dissatisfied' and 10 means 'completely satisfied'. Hence, we can match data for life satisfaction in year *t* with data on expectations about life satisfaction in year *t* expressed in year *t-5*, for each year *t* from 1996 until 2009 and for each individual that is present in both periods. By computing the difference between expectations and realizations, we can understand whether each individual’s current life satisfaction is below, in line with, or above the level he or she was aspiring to five years before.

**Fig 1:**
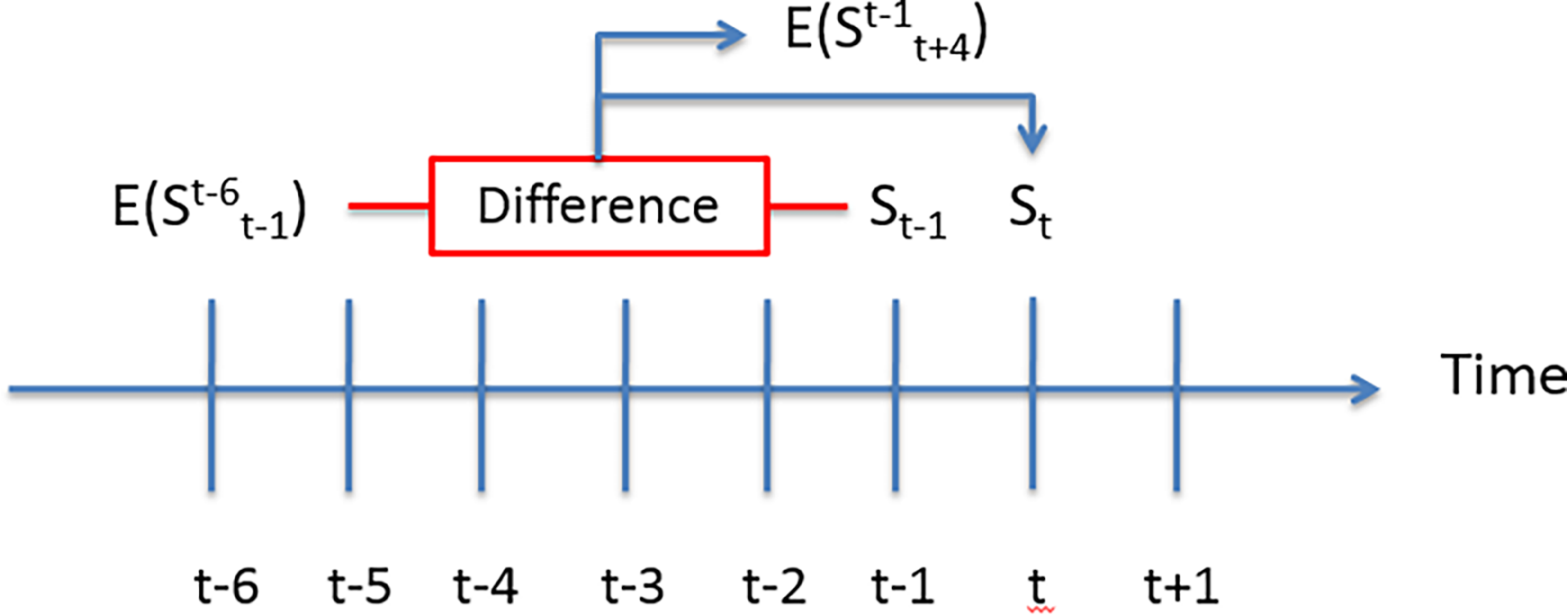
Timing of the analysis. Timing of our analysis of the consequences of positive and negative affective forecasting errors on future life satisfaction levels and on life satisfaction expectations.

Let the level of life satisfaction at period *t* be S_t_, and let life satisfaction expectations for period *t* expressed in period *t-5* be $E[S_{t}^{t-5}]$. We consider individual life satisfaction expectations to be unmet, met, or beaten if $S_{t}<E[S_{t}^{t-5}],\;S_{t}=E[S_{t}^{t-5}]$, or $S_{t}>E[S_{t}^{t-5}]$, respectively. We thus consider expectations to be beaten when individuals commit a positive affective forecasting error, met when there is no forecasting error, and unmet if the forecasting error is negative. Given our data, we are going to evaluate the effect of a positive or a negative difference between S_t−1_ and $E[S_{t-1}^{t-6}]$, our treatment variables, on the subsequent life satisfaction realization, S_t_, and on $E[S_{t+4}^{t-1}]$, the life satisfaction expectations expressed at time *t-1*. As a consequence, we consider only individuals for whom we observe S_t_, $E[S_{t+4}^{t-1}],$ S_t−1_ and $E[S_{t-1}^{t-6}]$, restricting our sample to years *t = 1997*, *…*, *2005*. The timing of our analysis is reported in Fig 1.

Our choice of comparing expected and realized emotional states is consolidated in the psychological literature studying validity and precision of affective forecasting, namely how individuals formulate predictions about their future 'feelings' (for an extensive survey, [26]). For instance, by comparing–as we do–expected and realized feelings, this literature has highlighted that while people are particularly skilled in predicting the valence (either positive or negative) and the emotional specificity (the specific nature of the future emotional reaction) of future feelings, they are less accurate in predicting the intensity and duration of these emotional states. More related to our case, several previous studies have already used the SOEP matched life satisfaction expectations-realizations data that we also exploit. One example is Frijters et al. [46], who show that East Germans overshoot their happiness expectations about the 1989 German unification. The second is Abolhassani and Alessie [47], who show that unemployed individuals expect to be less satisfied with life than they will actually be in the future, while the same does not hold for retirees. Third, Schwandt [29] shows that the observed U-shaped age profile of life satisfaction can be explained by unmet targets. Similarly, Lang et al. [48] study age-differential accuracy in anticipation of future life satisfaction. Finally, [49] show that people systematically mis-predict the impact of life events such as marriage, widowhood, unemployment and disability on subjective well-being, and that this is partly driven by unforeseen adaptation. While most of these studies focus on the determinants of the mismatch between expectations and realizations, we are instead interested in its effect on future life satisfaction, and we explicitly allow for the interplay between achievements and expectations of life satisfaction.

Using data from the Gallup World Poll in which people self-report evaluative well-being today and five years from now by answering to a standard Cantril ladder question, Deaton [50] shows that, in spite of repeated evidence to the contrary, people consistently predict they will be better off in the future. The gap between future and current well-being diminishes with age, and in rich countries, becomes negative among the elderly.

#### Demographic characteristics and other controls

We also make use of information on age, that enters in our model as a quadratic; interview year dummies (to avoid issues of multicollinearity due to age-period-cohort under-identification, we group together the first two years, i.e. 1997 and 1998); a variable counting the number of evaluations of life satisfaction expressed by the individual until any given year, that we call “evaluation experience” and that controls for learning effects; a dummy for residing in East Germany; years of education; employment status (dummies for full- or part-time employment, unemployment, retirement–the excluded category being out of the labor force); civil status (dummies for being married, divorced, widowed, the excluded category being single); number of children; objective health indicators (dummies for any doctor visits or any overnight hospital stay in the previous year, a dummy for having a statement of disability); net household income. Our control variables are measured at time t-6, when the affective forecasts were expressed. In addition, to take care of the improving or deteriorating trajectories in objective circumstances of individuals between the time of the formulation of expectations and the subsequent realization of life satisfaction, we also explicitly include variables indicating the experience of the event measured by each of the control variables between these two time periods. To give an example, we construct a dummy equal to 1 if an individual experienced any episode of unemployment between *t-5* and *t-1*. We proceed analogously for all other covariates, with the exception of age–as its change is mechanical–and survey year and evaluation experience, that we measure at time *t*. In addition, since education and household income are continuous variables, we simply control for the level of these two variables at *t-6* and *t-1*. Finally, for number of children we include whether any new child is born between *t-5* and *t-1*.

#### Income expectations

In an extension of our main analysis, we also use information about the self-reported probability of having a pay rise within the next two years, that we match with information on labor income changes for the corresponding time span. We consider income expectations to be “unmet” if individuals expected a pay rise with a positive probability and did not experience a positive change in labor income, and “beaten” if they did not expect a pay rise and instead got a positive labor income change. Similarly to the previous case, in this analysis we will consider how (not) getting a pay rise expected at time *t-3* for *t-1* affects life satisfaction levels at time *t* and life satisfaction expectations at time *t-1*.

#### Sample selection

The final sample for our main analysis is composed of an unbalanced panel of 75,231 observations for 13,431 individuals. This stems from the following sample selection rules:

1. Keep only time-*t* observations for years 1997–2005 (178,592 observations–initial sample)
2. Keep only observations with non-missing data about life satisfaction at *t* (178,167 observations).
3. Keep only observations with non-missing data about life satisfaction expectations expressed at t-6 for t-1 (85,342 observations).
4. Keep only observations with non-missing data about life satisfaction at *t-1* (83,947 observations)
5. Keep only observations with non-missing data about life satisfaction expectations expressed at *t-1* for *t+4* (82,998 observations)
6. Keep only observations with non-missing data about any other covariate used in the analysis (75,231 observations)

Our sample is smaller for the income expectations analysis, as pay rise expectations were consistently asked only to employed individuals interviewed in two specific years within our sample (*t-3 = 1999 and 2001*). The final sample in this case is composed of 12,105 observations for 8,362 individuals.

### Descriptive statistics

Descriptive statistics for our sample are shown in Table 1. Close to 52% of individuals in our sample are females, average age at the time when the expectations about life satisfaction is expressed is 43 years, 27% of our sample lives in East Germany, and the average years of education is 11.4. In terms of employment status, 62% of the sample is employed full-time, 3% is employed part-time, 7% is unemployed and 9% is retired. The average log net household income is 7.53 points. The distribution of civil status is as follows: 62% is married, 6% is divorced and 5% is widowed. The average number of children is 0.71. In the year before the interview, 58% of the sample went to the doctor at least once and 10% experienced an overnight hospital stay, while 12% of the sample has a disability statement. Descriptive statistics for the evolution of these variables between t-6 and t-1 are also reported in Table 1, where we also report the descriptive statistics for unmet and beaten income expectations, that we use in an extension to our main analysis. Finally, individuals carried out an average of 4 evaluations before expressing S_t_.

**Table 1:** Descriptive statistics.

	Mean	Standard Deviation
Age_t-6_	42.9	15.6
Female	0.52	0.50
East Germany_t-6_	0.27	0.45
Years of education_t-6_	11.4	2.46
Employed full time_t-6_	0.62	0.49
Employed part time_t-6_	0.03	0.18
Unemployed_t-6_	0.07	0.25
Retired_t-6_	0.09	0.29
log(Net household income)_t-6_	7.53	0.49
Married_t-6_	0.62	0.49
Divorced_t-6_	0.06	0.24
Widowed_t-6_	0.05	0.21
Number of children _t-6_	0.71	0.99
Any doctor visit_t-6_	0.58	0.49
Any overnight hospital stay_t-6_	0.10	0.30
Disability statement _t-6_	0.12	0.32
S_t_	6.75	1.77
$E[S_{t+4}^{t-1}]$	6.78	1.94
$S_{t-1}-E[S_{t-1}^{t-6}]$	-0.24	2.00
Unmet life satisfaction expectations	0.43	0.49
Beaten life satisfaction expectations	0.31	0.46
Evaluation experience_t_	4.03	2.28
Ever lived in East Germany between t-5 and t-1	0.28	0.45
Ever been employed full time between t-5 and t-1	0.72	0.45
Ever been employed part time between t-5 and t-1	0.10	0.30
Ever been unemployed between t-5 and t-1	0.16	0.37
Ever been retired between t-5 and t-1	0.15	0.36
Ever been married between t-5 and t-1	0.74	0.44
Ever been divorced between t-5 and t-1	0.10	0.30
Ever been widowed between t-5 and t-1	0.07	0.25
Any child born between t-5 and t-1	0.13	0.34
Any doctor visit between t-5 and t-1	0.94	0.24
Any overnight hospital stay between t-5 and t-1	0.37	0.48
Ever had a disability statement between t-5 and t-1	0.19	0.39
Years of education_t-1_	11.6	2.50
log(Net household income)_t-1_	7.62	0.50
Unmet income expectations	0.13	0.33
Beaten income expectations	0.36	0.48

*Notes*: the table reports descriptive statistics for the variables used in our empirical analysis. The number of observations is 75,231. Beaten and unmet income expectations are computed in the sub-sample of employed individuals present in *t-3 = 1999 and 2001*. The number of observations in this subsample is 12,228.

**Fig 2:**
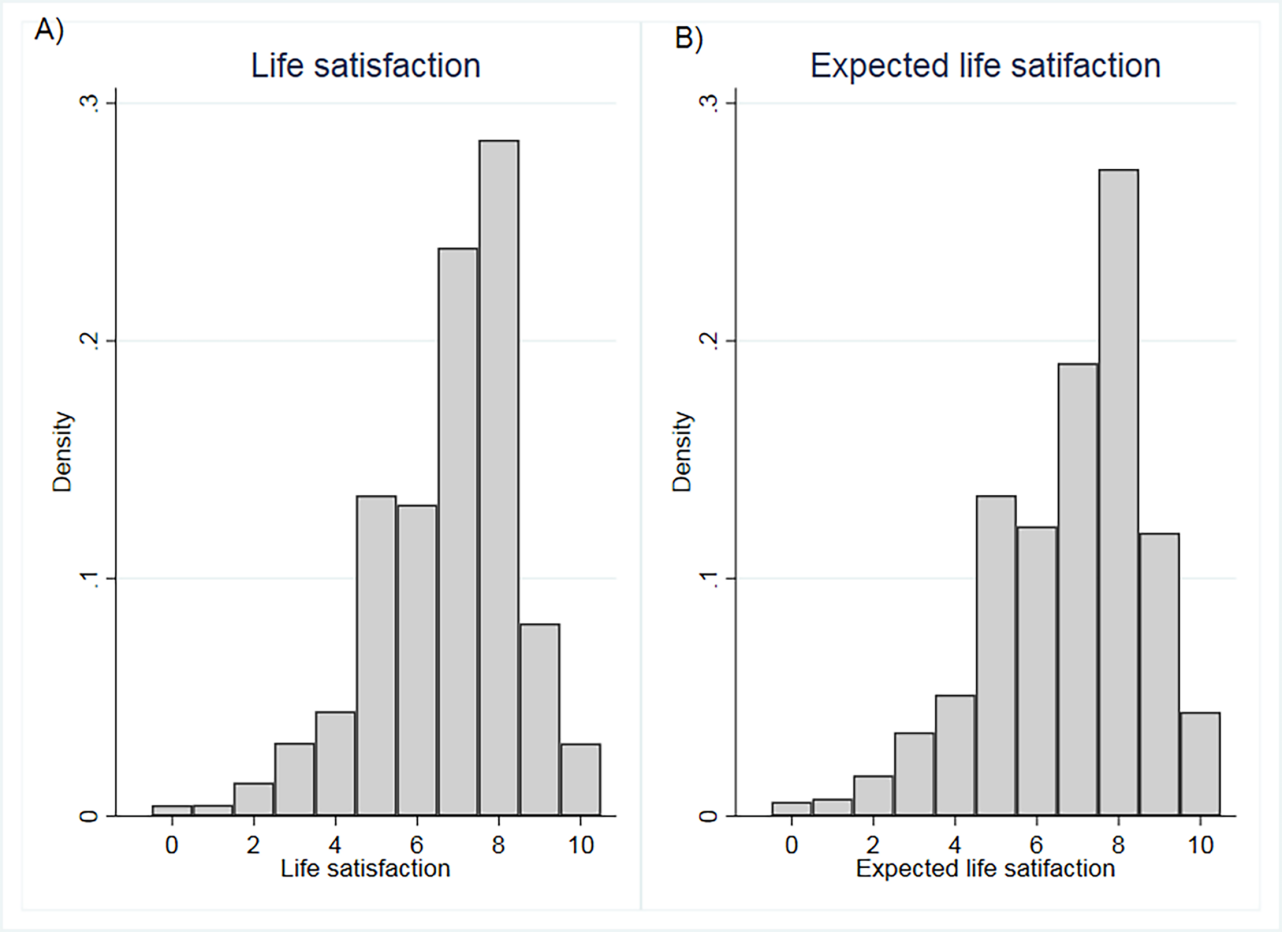
Empirical distributions of S_t_ and $E[S_{t+4}^{t-1}]$. Empirical distributions of S_t_ (A) and $E[S_{t+4}^{t-1}]$ (B) in our sample. The number of observations is 75,231.

Our main outcome variable is self-reported life satisfaction at time t, S_t_. It has an average of 6.75, and its standard deviation is equal to 1.77. Our second outcome variable is $E[S_{t+4}^{t-1}]$, that has a mean of 6.78 and a standard deviation of 1.94. For descriptive purposes, the histograms of the distributions of these two variables are respectively reported in Panels A and B of Fig 2.

**Fig 3:**
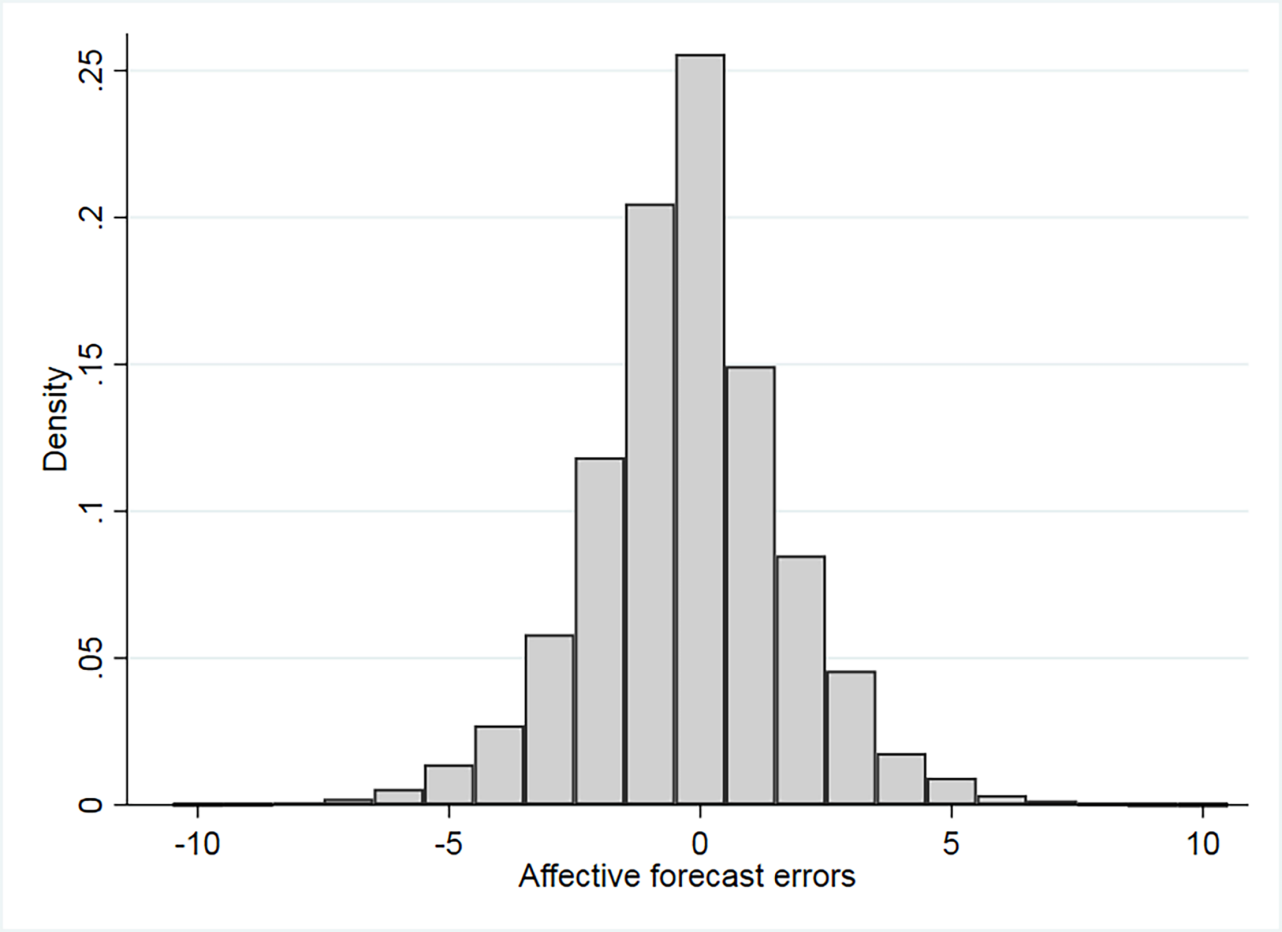
Empirical distribution of the affective forecasting error $S_{t-1}–E[S_{t-1}^{t-6}]$. Empirical distributions of the affective forecasting error $S_{t-1}—E[S_{t-1}^{t-6}]$ in our sample. The number of observations is 75,231.

We characterize positive and negative affective forecasting errors with two dummy variables, Unmet_i,t−1_ and Beaten_i,t−1_. These are equal to one for a negative or a positive affective forecasting error, i.e. a negative or a positive difference between S_t−1_ and $E[S_{t-1}^{t-6}]$. The average of this difference is equal to -0.24, with a standard deviation of 2.00. The histogram of this variable is reported in Fig 3, and confirms that the distribution of affective forecasting errors is skewed to the left. Overall, there are 43% of cases unmet expectations and 31% of cases of beaten expectations. Therefore, individuals formulate correct affective forecasts in 26% of cases.

The correlation between S_t−6_ and $E[S_{t-1}^{t-6}]$ is positive and equal to 0.68. This means that, on average, individuals with higher baseline life satisfaction have a tendency to express higher expectations about future life satisfaction. One way to interpret this very high correlation could be that individuals have adaptive expectations are adaptive, that is, that $E[S_{t-1}^{t-6}]=S_{t-6}$ + *e*, where *e* is a random error term. Indeed, under the assumption that individuals have fully adaptive expectations, in a robustness test we also substitute $E[S_{t-1}^{t-6}]$ with S_t−6_ when we compute the mismatch variables, and find comparable results (detailed estimation outcomes are available from the authors). On the other hand, although still positive, the correlation between S_t−6_ and S_t−1_ is lower, at 0.45. Table 2 shows the distribution of the beaten and unmet variables by S_t−6_, the level of life satisfaction when the expectations were expressed. The pattern that emerges from this analysis is not surprising: given their tendency to express high expectations, individuals with progressively higher levels of baseline life satisfaction are more likely to experience unmet expectations, and vice-versa. Hence, individuals with unmet expectations are positively selected with respect to their baseline life satisfaction, and vice-versa.

**Table 2:** Distribution of beaten and unmet expectations by the baseline level of life satisfaction S_t-6_.

S_t-6_	% beaten	% unmet
0	0.64	0.18
1	0.64	0.22
2	0.62	0.25
3	0.55	0.28
4	0.50	0.33
5	0.47	0.30
6	0.39	0.39
7	0.31	0.42
8	0.23	0.46
9	0.18	0.57
10	0.16	0.57

*Notes*: the table reports the distribution of Beaten and Unmet expectations by the baseline level of life satisfaction St-6. The number of observations is 75,231.

To understand the patterns of beaten and unmet expectations by other individual characteristics at the time when expressing the forecast, Table 3 reports their distribution according to age (below or above the median of 43 years), employment status (full-time employed vs. other), civil status (married vs. other), and overall health (disabled or not). The message delivered by this Table is similar to the one for baseline life satisfaction: likely because of their higher initial expectations, individuals with initially better conditions (younger, full time employed, married, not disabled) are the ones who are more likely to experience unmet and less likely to experience beaten expectations–suggesting positive selection into unmet expectations, and vice-versa. However, the magnitude of these differences is rather small.

**Table 3:** Distribution of beaten and unmet expectations by a set of observable characteristics at t-6.

	% beaten	% unmet
Age		
Less than 43 years	25%	50%
43 years or more	38%	36%
Full-time employed		
Yes	29%	45%
No	35%	40%
Married		
Yes	29%	46%
No	33%	41%
Disabled		
Yes	32%	43%
No	31%	43%

*Notes*: The number of observations is 75,231.

Finally, in Table 4 we provide a descriptive outlook of the relationship between changes in some objective circumstances and unmet/beaten expectations. Following Odermatt and Stutzer [49], we report the distribution of beaten and unmet expectations for individuals who:

- were single at *t-6* and got married between *t-6* and *t-1*—or not
- were married at *t-6* and became widowed between *t-6* and *t-1—*or not
- were full-time employed at *t-6* and experienced unemployment between *t-6* and *t-1*—or not
- were not disabled at *t-6* and became disabled between *t-6* and *t-1*—or not

**Table 4:** Distribution of beaten and unmet expectations for individuals becoming married, widowed, unemployed and disabled between t-6 and t-1.

	% beaten	% unmet
Single at t-6, becomes married		
Yes	25%	48%
No	26%	49%
Married at t-6, becomes a widow		
Yes	36%	43%
No	33%	41%
Employed at t-6, experiences unemployment		
Yes	28%	52%
No	29%	44%
Not disabled at t-6, becomes disabled		
Yes	34%	44%
No	31%	43%

*Notes*: The number of observations is 69,197.

As one would expect, those staying single, experiencing widowhood, unemployment and disability are more likely to have unmet expectations. The opposite does not hold, however, as in all cases except for unemployment these groups are also the ones more likely to experience beaten expectations. This is likely happening because these groups also have lower–and therefore easier to beat–expectations. Again, however, the differences are in most cases small in magnitude.

All in all, these descriptive facts highlight that–to understand the consequences of positive and negative affective forecasts on future life satisfaction—it is important to take into account of differences in individual characteristics at the time of the forecast and their changes over time, as these are likely correlated with both affective forecasting errors and life satisfaction.

### Empirical model

The aim of our empirical analysis is to provide first-pass empirical evidence on whether there are differential correlations between last-period unmet or beaten life satisfaction targets and one-period-ahead life satisfaction. We also assess whether there is a correlation between last-period unmet or beaten life satisfaction targets and last-period life satisfaction expectations for the future—contemporaneously with the realization of the met/unmet variable.

To do that, we estimate two panel-data equations, the first depicting the association between positive and negative affective forecasting errors and the actual level of life satisfaction and the second describing the correlation between well-being expectations and hedonic failures and successes.

The estimation of two different models for life satisfaction and for the expectations about life satisfaction is motivated by the findings by Frijters et al. [46] and Schwandt [29], who have shown that the differences between expectations and realizations of life satisfaction can be systematically predicted by information that is available at the time of the forecast–namely age and educational level–providing empirical evidence against the hypothesis of rational expectations [51] for life satisfaction.

The main difficulty with the interpretation of our estimated associations between affective forecasting errors and life-satisfaction concerns unobserved heterogeneity. First, comparability of self-reported life satisfaction across different individuals is hampered by issues of differential item functioning [52], as the interpretation of a life satisfaction scale may differ across individuals [53]. Furthermore, latent traits of individuals that are constant over time may determine both their life satisfaction levels and their propensity to report high or low expectations about future life satisfaction. Finally, as shown in the previous section, the omission of individual traits that change at the same time as the beaten/unmet expectation patterns could lead us to estimate biased associations between positive/negative forecasting mismatches and subsequent life satisfaction levels. For instance, a negative shock that has a long lasting effect on life satisfaction–like the onset of disability–could decrease life satisfaction both in the current *and* in the future period. If not properly controlled for, these persistent shocks would generate a spurious negative association between unmet expectations and one-period-ahead life satisfaction.

We solve issues of time-invariant unobserved heterogeneity using fixed effects panel data models, that allow us to purge our estimate from time-invariant individual traits. Assuming that reporting styles are constant within individuals and over time [54], inclusion of individual fixed effects also solves issues of differential item functioning. On the other hand, time-invariant covariates like gender and country of birth are absorbed by the individual fixed effects. We also control for a rich battery of individual time-varying observable covariates, described above and measured at *t-6* –when expectations were expressed. These controls help us to partial out the heterogeneity in observable individual characteristics *at the time* of formulating expectations. Additionally, to explicitly take into account that positive or negative affective forecasting errors may be actually related to an underlying *improving or deteriorating trend in objective circumstances*–as described by the disability example above–we also include controls for the evolution of these same covariates between *t-6* and *t-1* –when life satisfaction is realized. Although we would never be sure that we have included all potential sources of spurious correlation, we are confident that our set of controls is very comprehensive, and that it includes the most relevant life events that have been proven to be related to life satisfaction by previous empirical literature [55]. Even after including all the controls at *t-6* and their evolution between *t-6* and *t-1*—negative unobservable shocks affecting well-being can be more persistent than positive ones, which would make individuals have both unmet expectations *and* lower life satisfaction in the next period, but not the other way around. This is a remaining empirical difficulty that we can hardly overcome. Therefore, we will interpret all our results in terms of associations, and avoid to give causal interpretations to the estimated coefficients. Still, in the robustness section we discuss a placebo exercise that shall help to dispel this concern.

Formally, we estimate the following system of 2 equations:

$$\begin{array}{c} S_{i,t}=\alpha_{i}^{1}+\beta_{1}^{1}{Unmet}_{i,t-1}+\beta_{2}^{1}{Beaten}_{i,t-1}+X_{i,t-6}^{\prime }\gamma^{1}+∆X_{i,t-1}^{\prime }\theta^{1}+\xi_{i,t}^{1}\\ {E[S}_{i,t+4}^{t-1}]=\alpha_{i}^{2}+\beta_{1}^{2}{Unmet}_{i,t-1}+\beta_{2}^{2}{Beaten}_{i,t-1}+X_{i,t-6}^{\prime }\gamma^{2}+∆X_{i,t-1}^{\prime }\theta^{2}+\xi_{i,t}^{2} \end{array}$$

The first equation is related with life satisfaction levels at time *t*, while the second regards life satisfaction expectations at time *t-1*. In each equation, the α_i_ are individual fixed effects, *Unmet*_*t*−1_ and *Beaten*_*t*−1_ are two dummy variables defined above for unmet and beaten expectations (the reference group being meeting one's expectations), the vectors *X*_*t*−6_ and ΔX_i,t−1_ respectively include the time varying covariates measured at t-6 and their evolution up to t-1, as described above. Finally, $\xi_{i,t}^{j},j=1,2$ represent an error term: since the same respondent appears in our data multiple times, we always cluster standard errors at the individual level. We estimate the system depicted above equation-by-equation: since the same right-hand side variables appear in both models, joint estimation would lead to equivalent estimates [56]. Finally, we have performed cluster-robust Hausman tests to verify the plausibility of fixed vs. random effects models, and the tests always reject the random effects specification with p < .01.

## Results

### Main results

Our main empirical results are presented in Table 5, where we report the estimates of coefficients $\beta_{1}^{1},\beta_{2}^{1},\beta_{1}^{2}$ and $\beta_{2}^{2}$ obtained from our data. The first two columns show results for S_t_ and $E[S_{t+4}^{t-1}]$ when we do not include the controls in vectors *X*_*t*−6_ and ΔX_i,t−1_, while the last two columns show the same results when these controls are included. The estimated coefficients for *Unmet*_*t*−1_ and *Beaten*_*t*−1_ do not change across the two blocks of columns. This fact suggests that the associations that we pin down are robust to the confounding effect of the covariates included in the model. Looking at Column 1 and 3, we find that there is a negative correlation between the level of life satisfaction in the next period, S_t_, and failing to meet life satisfaction expectations (without controls: $\beta_{1}^{1}$ = -0.073, p < 0.01; with controls: $\beta_{1}^{1}$ = -0.071, p < 0.01), while there is no correlation between going beyond one's expectations and life satisfaction (without controls: $\beta_{2}^{1}$ = 0.019, p > 0.1; with controls: $\beta_{2}^{1}$ = 0.016, p > 0.1). This evidence confirms the asymmetric relationship between affective forecasting errors and life satisfaction highlighted in the model. We also test for equality of magnitude of the coefficients related with *Unmet*_*t*−1_ and *Beaten*_*t*−1_ (H_0_: $\beta_{1}^{1}=-\beta_{2}^{1}$) and reject the null with a p-value of 0.02.

**Table 5:** Association between unmet and beaten life satisfaction expectations, S_t_ and $E[S_{t+4}^{t-1}]$.

	(1)	(2)	(3)	(4)
Outcome variable:	S_t_	$E[S_{t+4}^{t-1}]$	S_t_	$E[S_{t+4}^{t-1}]$
*Unmet l*.*s*.*exp*._*t*−1_	-0.073***	-0.646***	-0.071***	-0.643***
	(0.014)	(0.014)	(0.014)	(0.014)
*Beaten l*.*s*.*exp*._*t*−1_	0.019	0.475***	0.016	0.473***
	(0.014)	(0.015)	(0.014)	(0.014)
Individual FE	Yes	Yes	Yes	Yes
Age quadratic	Yes	Yes	Yes	Yes
Year FE	Yes	Yes	Yes	Yes
Covariates	No	No	Yes	Yes
Observations	75,231	75,231	75,231	75,231
Individuals	13,431	13,431	13,431	13,431

*Notes*: All models control for individual fixed effects, a quadratic polynomial in age and year dummies. Models in Columns (3) and (4) also control for the covariate vectors *X*_*t*−6_ and ΔX_i,t−1_, described in the text. Number of observations and individuals stated at the bottom of each column. Robust standard errors clustered at the individual level in parentheses.

*** p<0.01

** p<0.05

* p<0.1.

As suggested by the theoretical model, one possible mechanism behind this result could be asymmetric rescaling of expectations as a result of beaten and unmet targets. We investigate this possibility in Column 2 and 4, where our dependent variable is the life satisfaction benchmark people set for the future, $E[S_{t+4}^{t-1}]$, after reporting S_t−1_. We find that expectations are positively correlated to positive forecasting errors (without controls: $\beta_{2}^{2}$ = 0.475, p < 0.01; with controls: $\beta_{2}^{2}$ = 0.473, p < 0.01), and negatively correlated to unmet expectations (without controls: $\beta_{1}^{2}$ = -0.646, p < 0.01; with controls: $\beta_{1}^{2}$ = -0.643, p < 0.01). In line with our theoretical framework, the previous findings can be rationalized as follows: although the estimated correlation between past beaten expectations and current expectations for the future is (in absolute value) smaller than in case of unmet expectations (the difference is statistically significant with p<0.01), in the first case the adjustment in expectations (the third channel by which affective forecasting errors affect life satisfaction in the model) is strong enough to cancel out the potential positive effects of beaten expectations (that consists of the joint effect of the first two channels) on life satisfaction. Instead, in the second case it is not, and individuals persistently bear the negative emotional consequences of not achieving expectations.

### Extensions

Satisfaction with life as a whole can be described as a weighted average of satisfaction with several economic and non-economic aspects of life [38]. It would therefore be interesting to understand whether beaten or unmet expectations about economic or non-economic aspects of life are driving our findings. In an attempt to answer this question, we replicate our main analysis focusing on beaten and unmet expectations about *income*, defined as described above. These regressions are structured like our main analysis, but include among the controls a dummy indicating whether individuals were expecting or not a pay rise with positive probability and the realized levels of individual labor income at *t-3* and *t-1*.

Of course, we would like to carry out a similar analysis about other economic and non-economic life domains (such as employment, occupation choice, family relations, health, …) but our data only provide consistent longitudinal information about expectations and realizations of this specific dimension of life.

Results are reported in Table 6. On the one hand, there is weakly significant (0.1 < p < 0.05) evidence that the “satisfaction treadmill” is at work for *income* expectations, as there is a positive correlation between higher life satisfaction expectations and surpassing their *income* expectations, but not vice-versa (without controls: $\beta_{1}^{2}$ = 0.013, p > 0.1 and $\beta_{2}^{2}$ = 0.206, 0.1 < p < 0.05; with controls: $\beta_{1}^{2}$ = 0.024, p > 0.1 and $\beta_{2}^{2}$ = 0.197, 0.1 < p < 0.05). On the other hand, neither positive nor negative mismatches between *income* expectations and realizations correlate with future life satisfaction (without controls: $\beta_{1}^{1}$ = 0.028, p > 0.1 and $\beta_{2}^{1}$ = 0.078, p > 0.1; with controls: $\beta_{1}^{1}$ = 0.004, p > 0.1 and $\beta_{2}^{1}$ = 0.096, p > 0.1).

**Table 6:** Association between unmet and beaten income expectations, S_t_ and $E[S_{t+4}^{t-1}]$.

	(1)	(2)	(3)	(4)
	S_t_	$E[S_{t+4}^{t-1}]$	S_t_	$E[S_{t+4}^{t-1}]$
*Unmet income exp*._*t*−1_	0.028	0.013	0.004	0.024
	(0.104)	(0.116)	(0.104)	(0.116)
*Beaten income exp*._*t*−1_	0.078	0.206*	0.096	0.197*
	(0.108)	(0.115)	(0.109)	(0.114)
Individual FE	Yes	Yes	Yes	Yes
Age quadratic	Yes	Yes	Yes	Yes
Year FE	Yes	Yes	Yes	Yes
Basic covariates	Yes	Yes	Yes	Yes
Other covariates	No	No	Yes	Yes
Observations	12,105	12,105	12,105	12,105
Individuals	8,362	8,362	8,362	8,362

*Notes*: All models control for individual fixed effects, a quadratic polynomial in age, year dummies, log of income at t-3 and t-1, and for the income expectations expressed at t-3 for t-1. Models in Columns (3) and (4) also control for the covariate vectors *X*_*t*−6_ and ΔX_i,t−1_, described in the text. Number of observations and individuals stated at the bottom of each column. Robust standard errors clustered at the individual level in parentheses.

*** p<0.01

** p<0.05

* p<0.1.

### Robustness tests

In S1 File, we present some sensitivity tests to our main empirical results, that we describe here. For all robustness tests we report only the estimated coefficient for the models including all covariates–as in Columns (3) and (4) of Table 5 –but results are unchanged when we exclude them–as in Columns (1) and (2) of Table 5.

First, in our main analysis we have used categorical indicators for beaten or unmet expectations. However, it is not granted that unmet and beaten expectations dummies represent reverse forecasting errors of the same strength. For example, it could be that positive forecasting errors represent on average greater absolute errors than negative forecasting errors. Therefore, as a robustness test we consider a specification where, instead of the dummies, we use a linear spline in the value of the difference between life satisfaction realizations and expectations, with a knot at a difference of zero. As shown in Table A in S1 File, the patterns we detect are analogous to the ones using the categorical indicators We tested for difference in slopes between the positive and the negative segments, and we can reject the null of equal trends with p<0.01 for both S_t_ and $E[S_{t+4}^{t-1}]$. We also experimented with non-linear specifications where–instead of including a linear spline–we have included dummies for each quartile of the positive-negative mismatch. Results (available from the authors) suggest that the linear specification provides a good approximation of the empirical relationships investigated.

Second, as shown in Table B in S1 File, the patterns we have shown are stable across the general population, as our findings are robust to dropping people aged 65+ from our sample, and we obtain similar results also when we split the sample between males and females or drop observations for which the value of expected life satisfaction is either 0 or 10, as individuals cannot fail to meet expectations equal to 0 and cannot beat expectations equal to 10.

As interview dates are not random–in Table C in S1 File we replicate our analysis and include as additional controls in our model the distance (in days) between *(i)* the date when S_t-1_ is realized and the date when E_t-6_(S_t-1_) was expressed and *(ii)* the date when S_t_ is realized and the date when S_t-1_ was realized (only in the model for S_t_). Although the number of observations differs because of missing data in interview dates, results are wholly consistent with our baseline. Results still hold if we use non-linear functions of these distances.

Third, since Schwandt [29] highlights how affective forecasting errors are more negative early in life and turn positive later on, we are particularly concerned that a kink in well-being around a threshold age may be driving our results. Although our baseline specification already controls for a quadratic trend in age–therefore allowing for a non-linear relationship–to address this concern we also show in Table D in S1 File that our results are not qualitatively affected when we allow for a fully flexible specification for age, that includes either age dummies or age-by-gender dummies. In addition, results for this more demanding specification are unchanged even when we only focus on working age (25–65) respondents.

A fourth issue about our design concerns expectation updating. In fact, our main explanatory variables depend on the differences in achieved life satisfaction and the expected life satisfaction measure *from five years before*. By using the latter as our benchmark, we are implicitly assuming that individuals do not adapt their expectations in the meantime. However, individuals may get feedback on their expectations and achieved well-being every year (every second, actually) and adjustments of expectations may happen during the five year period, rendering our chosen benchmark outdated. To understand whether expectation updating can invalidate our empirical conclusions, we proceed in two steps. First, we rely on SOEP data for year 2008, when respondents were asked to express expectations about life satisfaction in both 1 and 5 years in the future. In this data, the correlation between the two variables is equal to 0.83. Indeed, for 85% of respondents the expectation for t+1 is in a 1-point interval around the expectation for t+5, and the two expectations correspond exactly for 55% of respondents. This evidence suggests that–when thinking about expected life satisfaction—respondents may not have in mind a precise time horizon, but think about a generic “future” period. Having verified this first point, we can then go back to our original data and match expectations expressed a *t-2* for five years in the future—$E[S_{t+3}^{t-2}]$—to life satisfaction in *t-1*, and repeat our analysis. If expectation adjustments were a major issue, then using our specification based on the 5-year lagged expectation ($E[S_{t-1}^{t-6}])$ or the one based on 1-year lagged expectations ($E[S_{t+3}^{t-2}])$ should produce different results. Instead, if the two specifications lead to similar results then the role of expectation adjustments is minor in explaining our findings. Table E in S1 File reports results of our main analysis when we use 1-year lagged expectations (the different number of observations is due to missing values). Results are fully comparable to those considering the 5-year lag, confirming that–if anything–expectations updating plays a minor role in explaining our findings.

As already discussed in the previous pages, a final issue about our interpretation of the negative association between unmet expectations and subsequent life satisfaction realizations concerns the possibility that—even after including all the controls at *t-6* and their evolution between *t-6* and *t-1*—negative unobservable shocks affecting well-being can be more persistent than positive ones, which would make individuals have both unmet expectations *and* lower life satisfaction in the next period, but not the other way around. This is a remaining concern for the interpretation of our estimated associations as causal effects. To indirectly dispel this concern, we have devised a placebo test. On the one hand, if the effect of unmet expectations was only due to a stronger persistence of unobservable negative shocks to life satisfaction, then it should be also detected when the realized level of life satisfaction is matched to a randomly drawn expectation, as the only genuine determinant of a possible mismatch in this simulated setup is the realized level of life satisfaction. On the other hand, since a randomly assigned expectation is surely uncorrelated with life satisfaction, we should find a zero unmet effect if this was not due to asymmetric persistence of shocks. To perform this test, we have carried out a set of 1,000 random permutations of the expectation target ($E[S_{t-1}^{t-6}]$) within our sample. For each permutation, we have computed the beaten and unmet variables comparing the randomly drawn target, $E[S_{t-1}^{t-6}],$ with the realized level of life satisfaction, S_t−1_, and we have re-estimated the model. Since our focus is on the persistency of negative shocks to life satisfaction, we only consider the equation for S_t_ in this analysis. Table F in S1 File reports the median $Unmet_{t-1}(\beta_{1}^{1})$ and $Beaten_{t-1}(\beta_{2}^{1})$ coefficients that results from the 1,000 permutations, with their respective empirical confidence intervals at the 5% level of significance. Since neither $\beta_{1}^{1}$ nor $\beta_{2}^{1}$ are statistically different from zero, this placebo test does not lend empirical support to the hypothesis that our uncovered asymmetric affective forecasting error effects are due to the fact that negative shocks to life satisfaction have stronger and more persistent effects than positive ones.

## Discussion and conclusion

Social scientists have theorized that subjective well-being is a relative concept, that also depends on the discrepancy between its realization and an expected level [19–21]. Our study develops a simple theoretical framework to discuss the matter, and reports preliminary empirical evidence in favor of this hypothesis.

We find that the discrepancy between expected and realized levels of life satisfaction *per se* is significantly correlated with the subsequent well-being level, even after controlling for individual characteristics and potential biases in reporting styles. By so doing, we provide the first empirical evidence on people's emotional response to beaten or unmet affective forecasts [26]. We have also put forward a number of additional research questions to qualify the uncovered correlations and understand the main mechanisms at work. First, we have investigated the asymmetric correlation of beaten and unmet expectations with subjective well-being. In this respect, our results suggest that while going beyond one’s expectations is uncorrelated with subjective well-being, becoming aware of a negative discrepancy between actual and expected well-being is significantly and negatively correlated with its subsequent realizations.

Second, our results are supportive of the idea that that individuals tend to revise their targets of subjective well-being along with achievements and failures: future expectations are positively correlated with beaten past expectations, and negatively correlated with unmet past targets. While the adjustment in expectations appears to be strong enough to cancel the correlation of beaten expectations with subjective well-being, results support the idea that individuals fail to entirely internalize unmet targets, thus exhibiting a higher emotional sensitivity to negative affective forecasting errors.

Third, in an attempt to characterize whether our results are due to mismatches in expectations and realizations about economic, non-economic or both domains of life, we replicate our analysis using mismatches between *income* expectations and realization. On the one hand, we find that surpassing income expectations is positively correlated with expectations about future subjective well-being, highlighting how beaten expectations about economic aspects of life matter in terms of shaping expectations. On the other hand, discrepancies between expected and realized *income* do not correlate with subjective well-being levels, therefore suggesting that negative mismatches between expectations and realizations on variables concerning *non-income* aspects of life are responsible for the uncovered negative correlation of negative forecasting errors with future well-being.

Our results suggest that the pursue of high expectations on subjective well-being—likely to be unmatched by future conditions—will hurt long-run life satisfaction. This finding also provides empirical support in favor of the link between unmet aspirations and subsequent happiness that was hypothesized by Schwandt [29]. In this vein, Van Dijk et al. [57] report experimental evidence showing that subjects deliberately tend to lower their expectations about obtaining a desired but uncertain outcome in order to avoid future disappointment. Low expectations on subjective well-being—likely to be beaten by subsequent realizations—will not pay-off either, as they would be adjusted upwards before leading to true gains in subjective well-being.

## Supporting information

S1 File Containing Tables A-F.
(DOCX)
