# Supplementary material for: Asymmetric affective forecasting errors and their correlation with subjective well-being
Source: PLoS One. 2018 Mar 7;13(3):e0192941. doi: 10.1371/journal.pone.0192941 (PMC5841766; doi:10.1371/journal.pone.0192941)
Supplement: S1 File — Containing Tables A-F. (DOCX) [file pone.0192941.s001.docx]

**Supporting information**

Table A. Alternative specification: spline - linear trends in positive and negative affective forecasting errors

|  | (1) | (2) |
| --- | --- | --- |
|  | $S_{t}$ | $E\left[ S_{t+4}^{t-1} \right]$ |
|  |  |  |
| *Positive affective forecasting error_t-1_ – linear trend* | 0.006 | 0.220*** |
|  | (0.006) | (0.007) |
| *Negative affective forecasting error_t-1_ – linear trend* | 0.035*** | 0.384*** |
|  | (0.006) | (0.007) |
|  |  |  |
| Individual FE | Yes | Yes |
| Age quadratic | Yes | Yes |
| Year FE | Yes | Yes |
| Covariates | Yes | Yes |
| Observations | 75,231 | 75,231 |
| Individuals | 13,431 | 13,431 |

*Notes*: All models control for individual fixed effects, a quadratic polynomial in age and year dummies, and for the covariate vectors $X_{t-6}$ and $\Delta X_{i,t-1}$, described in the text. Number of observations and individuals stated at the bottom of each column. Robust standard errors clustered at the individual level in parentheses. *** p<0.01, ** p<0.05, * p<0.1.

Table B Robustness tests – different subsamples

|  | (1) | (2) | (3) | (4) | (5) | (6) | (7) | (8) |
| --- | --- | --- | --- | --- | --- | --- | --- | --- |
|  | Age ≤ 65 | | Females | | Males | | 0<$E\left[ S_{t-1}^{t-6} \right]$<10 | |
|  | $S_{t}$ | $E\left[ S_{t+4}^{t-1} \right]$ | $S_{t}$ | $E\left[ S_{t+4}^{t-1} \right]$ | $S_{t}$ | $E\left[ S_{t+4}^{t-1} \right]$ | $S_{t}$ | $E\left[ S_{t+4}^{t-1} \right]$ |
|  |  |  |  |  |  |  |  |  |
| $Unme{t l. s. exp.}_{t-1}$ | -0.056*** | -0.613*** | -0.084*** | -0.656*** | -0.055*** | -0.631*** | -0.073*** | -0.647*** |
|  | (0.015) | (0.015) | (0.020) | (0.019) | (0.019) | (0.020) | (0.014) | (0.014) |
| $Beate{n l. s. exp.}_{t-1}$ | 0.020 | 0.451*** | 0.024 | 0.470*** | 0.007 | 0.476*** | 0.011 | 0.500*** |
|  | (0.016) | (0.015) | (0.020) | (0.019) | (0.020) | (0.020) | (0.014) | (0.014) |
|  |  |  |  |  |  |  |  |  |
| Individual FE | Yes | Yes | Yes | Yes | Yes | Yes | Yes | Yes |
| Age quadratic | Yes | Yes | Yes | Yes | Yes | Yes | Yes | Yes |
| Year FE | Yes | Yes | Yes | Yes | Yes | Yes | Yes | Yes |
| Covariates | Yes | Yes | Yes | Yes | Yes | Yes | Yes | Yes |
| Observations | 62,506 | 62,506 | 39,188 | 39,188 | 36,043 | 36,043 | 70,495 | 70,495 |
| Individuals | 11,652 | 11,652 | 6,962 | 6,962 | 6,469 | 6,469 | 13,199 | 13,199 |

*Notes*: All models control for individual fixed effects, a quadratic polynomial in age and year dummies, and for the covariate vectors $X_{t-6}$ and $\Delta X_{i,t-1}$, described in the text. Number of observations and individuals stated at the bottom of each column. Robust standard errors clustered at the individual level in parentheses. *** p<0.01, ** p<0.05, * p<0.1

Table C. Robustness tests - different functional forms for age

|  | (1) | (2) | (3) | (4) | | (5) | (6) | | (7) | (8) |
| --- | --- | --- | --- | --- | --- | --- | --- | --- | --- | --- |
|  | Quadratic age trend | | Age dummies | | | Age by gender dummies | | | Age by gender dummies  25 – 65 years sample | |
|  | $S_{t}$ | $E\left[ S_{t+4}^{t-1} \right]$ | $S_{t}$ | | $E\left[ S_{t+4}^{t-1} \right]$ | $S_{t}$ | | $E\left[ S_{t+4}^{t-1} \right]$ | $S_{t}$ | $E\left[ S_{t+4}^{t-1} \right]$ |
|  |  |  |  | |  |  | |  |  |  |
| $Unme{t l.s. exp.}_{t-1}$ | -0.071*** | -0.643*** | -0.071*** | | -0.643*** | -0.069*** | | -0.642*** | -0.052*** | -0.621*** |
|  | (0.014) | (0.014) | (0.014) | | (0.014) | (0.014) | | (0.014) | (0.015) | (0.016) |
| $Beate{n l.s. exp.}_{t-1}$ | 0.016 | 0.473*** | 0.013 | | 0.472*** | 0.014 | | 0.471*** | 0.020 | 0.452*** |
|  | (0.014) | (0.014) | (0.014) | | (0.014) | (0.014) | | (0.014) | (0.015) | (0.015) |
|  |  |  |  | |  |  | |  |  |  |
| Individual FE | Yes | Yes | Yes | | Yes | Yes | | Yes | Yes | Yes |
| Year FE | Yes | Yes | Yes | | Yes | Yes | | Yes | Yes | Yes |
| Covariates | Yes | Yes | Yes | | Yes | Yes | | Yes | Yes | Yes |
| Observations | 75,231 | 75,231 | 75,231 | | 75,231 | 75,231 | | 75,231 | 60,402 | 60,402 |
| Individuals | 13,431 | 13,431 | 13,431 | | 13,431 | 13,431 | | 13,431 | 11,231 | 11,231 |

*Notes*: All models control for individual fixed effects, a quadratic polynomial in age and year dummies, and for the covariate vectors $X_{t-6}$ and $\Delta X_{i,t-1}$, described in the text. Number of observations and individuals stated at the bottom of each column. Robust standard errors clustered at the individual level in parentheses. *** p<0.01, ** p<0.05, * p<0.1.

Table D. Robustness tests - Including the distance between interviews as additional control

|  | (1) | (2) |
| --- | --- | --- |
|  | $S_{t}$ | $E\left[ S_{t+4}^{t-1} \right]$ |
|  |  |  |
| $Unme{t l. s. exp.}_{t-1}$ | -0.065*** | -0.642*** |
|  | (0.014) | (0.014) |
| $Beate{n l. s. exp.}_{t-1}$ | 0.002 | 0.470*** |
|  | (0.014) | (0.014) |
|  |  |  |
| Individual FE | Yes | Yes |
| Age quadratic | Yes | Yes |
| Year FE | Yes | Yes |
| Covariates | Yes | Yes |
| Observations | 70,964 | 70,964 |
| Individuals | 12,836 | 12,836 |

*Notes*: All models control for individual fixed effects, a quadratic polynomial in age and year dummies, for the covariate vectors $X_{t-6}$ and $\Delta X_{i,t-1}$, described in the text, as well as for the distance (in days) between: (i) the date when St-1 is realized and the date when Et-6(St-1) was expressed; (ii) the date when St is realized and the date when St-1 was realized (only in the model for St). Number of observations and individuals stated at the bottom of each column. Robust standard errors clustered at the individual level in parentheses. *** p<0.01, ** p<0.05, * p<0.1

Table E. Robustness tests – Unmet and Beaten computed on the basis of the $S_{t-1}-E\left[ S_{t+3}^{t-2} \right]$affective forecasting error

|  | (1) | (2) |
| --- | --- | --- |
| Outcome variable: | $S_{t}$ | $E\left[ S_{t+4}^{t-1} \right]$ |
|  |  |  |
| $Unme{t l. s. exp.}_{t-1}$ | -0.046*** | -0.614*** |
|  | (0.014) | (0.014) |
| $Beate{n l. s. exp.}_{t-1}$ | -0.003 | 0.500*** |
|  | (0.015) | (0.014) |
|  |  |  |
| Individual FE | Yes | Yes |
| Age quadratic | Yes | Yes |
| Year FE | Yes | Yes |
| Covariates | Yes | Yes |
| Observations | 72,500 | 72,500 |
| Individuals | 13,086 | 13,086 |

*Notes*: Unmet and Beaten computed on the basis of the affective forecasting error $S_{t-1}-E\left[ S_{t+3}^{t-2} \right].$All models control for individual fixed effects, a quadratic polynomial in age and year dummies, and for the covariate vectors $X_{t-2}$ and $\Delta X_{i,t-1}$, described in the text and whose timing in this case is consistent with that of $E\left[ S_{t+3}^{t-2} \right]$. Number of observations and individuals stated at the bottom of each column. Robust standard errors clustered at the individual level in parentheses. *** p<0.01, ** p<0.05, * p<0.1.

Table F. Placebo test – randomly assigned expectations vs. truly realized life satisfaction

|  | (1) |
| --- | --- |
|  | $S_{t}$ |
|  |  |
| $Unme{t random l.s. exp.}_{t-1}$ | -0.003 |
|  | (-0.022;0.029) |
| $Beate{n random l.s. exp.}_{t-1}$ | -0.004 |
|  | (-0.044;0.05) |
|  |  |
|  |  |
| Individual FE | Yes |
| Year FE | Yes |
| Covariates | Yes |
| Observations | 75,231 |
| Individuals | 13,431 |

*Notes*: We report the median and the empirical confidence intervals at the 5% level of confidence for the coefficients of beaten and unmet expectations derived from re-estimating the model after each of 1,000 random permutations of the life satisfaction expectation $E\left[ S_{t-1}^{t-6} \right]$. The outcome is S_t_, and the specification is the same adopted in Column (3) of Table 5. Number of observations and individuals stated at the bottom of each column. *** p<0.01, ** p<0.05, * p<0.1.
